# *In silico* design of Ebola virus Glycoprotein antigenic peptides as vaccine candidates

**DOI:** 10.1371/journal.pone.0319496

**Published:** 2025-03-28

**Authors:** David Lara-Ramírez, Clara Esperanza Santacruz-Tinoco, Eva Ramón-Gallegos, José Esteban Muñoz-Medina

**Affiliations:** 1 Environmental Cytopathology Laboratory, Escuela Nacional de Ciencias Biológicas, Instituto Politécnico Nacional, Mexico CityMexico; 2 División de Laboratorios Especializados. Instituto Mexicano del Seguro Social, Mexico City, Mexico; Cholistan University of Veterinary and Animal Sciences, PAKISTAN

## Abstract

Ebola virus (EBOV) is a filovirus that causes severe hemorrhagic fever and has a fatality rate between 50 and 90%. The vaccines were developed against the Ebola Zaire species; therefore, it is necessary to develop vaccines against other species to control future outbreaks. The objective of this work was to obtain vaccine candidate peptides against different EBOV species through the use of bioinformatics programs and servers that allow glycoprotein (GP) to be analyzed. GP sequences of various EBOV species that did not present gaps or unspecified amino acids or that were repeated (same year, region and laboratory) were downloaded from the NCBI database. A consensus sequence was generated and used to determine vaccine candidate peptides, which were evaluated, through a combination of servers and molecular dynamics, for their ability to interact with B and T lymphocytes, toxicity, allergenicity, solvent exposure, glycosylation, antigenicity, and presence in mature GP. Five vaccine candidate peptides were identified, of which PEP4 had the best characteristics evaluated in this study. PEP4 may be a potential candidate for the development of an EBOV vaccine.

## Introduction

Ebola virus (EBOV) belongs to the Filoviridae family. Currently, there are six species: Zaire, (EBOV), Reston (RESTV), Sudan (SUDV), Taï Forest (TAFV), Bundibugyo (BDBV), and Bombali (BOMV) [1,2]. The virion contains negative-polarity RNA (approximately ~ 19.1 kb) and is approximately ~ 1400 nm long and 80 nm in diameter. Structurally, it contains 3 layers: the nucleocapsid, which measures between 20 and 30 nm in diameter and is composed of a nucleoprotein (NP), RNA polymerase (L), and viral genetic material; the helical capsid, which measures between 40 and 50 nm in diameter and is composed of the VP24, VP30, VP35 and VP40 proteins that, in addition to being part of the structure, serve as interferons and cofactors for the viral polymerase; and the viral envelope, which measures approximately 80 nm in diameter and contains the trimeric protein glycoprotein (GP), which participates in the adsorption process and modulates the host’s immune response [3,4].

Since 1976, when the first cases occurred in the Democratic Republic of the Congo [5], numerous outbreaks, which vary in the number of cases and percentage of fatalities, have been recorded. The most notable Ebola epidemic occurred between 2014 and 2016 in Sierra Leone, Liberia and Guinea, with more than 28,600 cases and 11,320 deaths [6]. There have been outbreaks since those in 2014 and 2016; the last one occurred in 2022 in Uganda, where 142 cases and 55 deaths were reported by SUDV [7].

The development of EBOV vaccines has been challenging mainly because of the mechanisms of evasion of the immune response of the virus and because laboratories with a specialized infrastructure are required to work directly with the virus.

Vaccination continues to be an efficient way to control and even eradicate various diseases in the world, for example, smallpox. By the end of 2022, there were 80 studies on the development of vaccines against EBOV, of which 63 have concluded based on data from ClinicalTrials.gov. Most vaccines that have reached advanced stages of research have used GP as a target site because it is exposed on the structure of the virus and because antibodies generated by people who manage to recover from the disease recognize this protein [8, 9, 10].

Most of EBOV vaccines have been specifically designed against Ebola Zaire, causing concern among health institutions about other species, such as Ebola Sudan, that represent a high risk to public health; consequently, the development of vaccines against other EBOV species or that show cross-reaction between EBOV species is important [11].

Currently, bioinformatics tools are being used to perform various analyses in the health area, and these tools have facilitated the processes of obtaining new drugs and peptides used in vaccines and for the molecular study of the evolution of noninfectious diseases [12].

Regarding vaccine design, the use of bioinformatics tools in the early stages of development is widely profitable because they allow a first approximation of interaction, affinity and specificity, even before carrying out the first tests directly with the pathogen [13,14].

For this reason, the aim of this study was to obtain antigenic peptides from a consensus GP sequence from sequences for GP in various EBOV species so as to identify vaccine candidates. The peptides were evaluated *in silico* in terms of their toxicity, allergenicity, exposure to the solvent and ability to be recognized by B and T lymphocytes, in addition to testing the binding strength between peptides and HLA by means of a docking technique. The results were used to select the peptides that could serve as vaccine candidates.

## Materials and methods

### Study design

**Fig 1:**
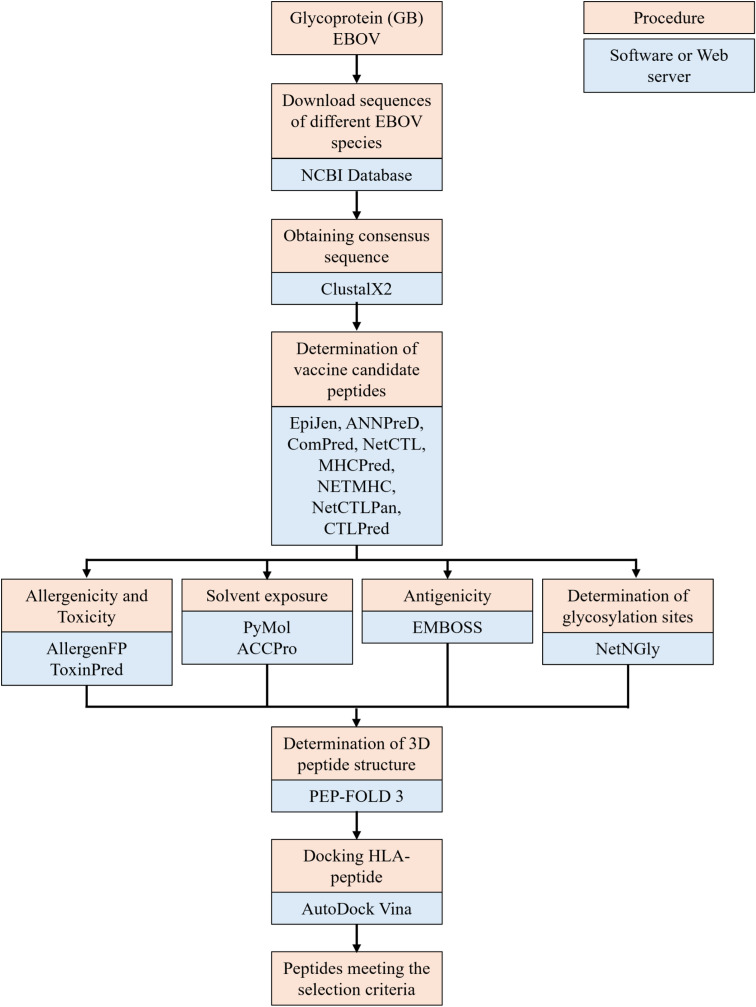
General scheme of the study. Steps followed for the selection and analysis of peptides.

A bioinformatic study was carried out in which complete sequences of GP in different EBOV species were downloaded from the NCBI database. Complete sequences were used to obtain a consensus GP sequence. Using various servers, the consensus sequence was used to determine which peptides of GP interact with B and T lymphocytes. By means of multiple alignment, those peptides that were found in a region with the highest frequency of sites identified within the consensus sequence were selected as vaccine candidates. Vaccine candidate peptides were analyzed using bioinformatics programs to evaluate toxicity, allergenicity, exposure to the solvent within the complete GP, glycosylation sites within GP, presence in mature GP, and the binding strength between each peptide and HLA. This process is shown in Fig 1.

### Obtaining the consensus sequence for EBOV GP

GP sequences for different EBOV species that had a length of 676 amino acids were downloaded from the NCBI database; only this length was used because 676 amino acids are the total size of this protein. Sequences with gaps or unspecified amino acids or that were repeated (same year, region and laboratory) were excluded. The included sequences were aligned using ClustalX2 V2.1 [15,16]. The consensus sequence was obtained using the WebLogo 3 server [17]. This sequence was used for peptide prediction.

### Prediction of antigenic peptides from EBOV GP

To identify antigenic peptides in the consensus sequence, the Immunomedicine Group server of Complutense University of Madrid, which has an accuracy of 75% in identifying linear peptides, was used [18]. To identify peptides that are recognized by T lymphocytes, EpiJen v1.0 [19,20] was used; the program employs multiple algorithms to identify peptides using a cutoff value of 5% for the best peptides identified for the output file, was used; this value is recommended by the server. ANNPreD, ComPred [21] and NetCTL [22,23], which are artificial neural networks (ANNs), were used to predict binding using a cutoff value of 0.5, which is the recommended efficiency weight because it provides optimal predictive performance. MHCPred v2.0 [24,25] was used to predict the affinity of peptides to class I and II MHCs and to the transporter associated with antigen processing (TAP); peptides with the highest -logIC50 values and with a prediction confidence of 1 were selected. NetMHC v4.0 [26], which uses an ANN-based method, was used to predict peptides recognized by different MHC alleles. NetCTLPan v1.1 [27,28], which uses multiple algorithms, and CTLPred [29,30], which uses a combination of ANN and SVM, were used to identify which peptides had the ability to interact with MHC type I molecules.

To identify peptides recognized by B lymphocytes, the BcePred server was used [31,32,33]; this server has the ability to predict continuous peptides based on physicochemical properties in a nonredundant database, and the precision varies from 52.92% to 57.53%. BepiPred v2.0 [34], which uses the random forest algorithm trained with annotated peptides from the structure of antigen-antibody proteins, was used. Discotopo v2.0 [35] was used to determine discontinuous peptides from the three-dimensional structure of the protein.

For the aforementioned servers, the HLA supertypes of human A2, A3 and B7 were used because they are present in approximately 86% of the world’s population [27]. After this process, peptides were selected for vaccine development.

### Selection of vaccine candidate peptides from the consensus sequence

Vaccine candidate peptides were selected using the following criteria: 1) present in regions where the greatest amount of peptides were aligned, as determined using the aforementioned servers [36] interact with B lymphocytes as well as with HLA supertypes A2, A3 and B7 of T lymphocytes.

### Allergenicity analysis of vaccine candidate peptides

This analysis was performed using the AllergenFP v1.0 server [37]; the amino acid sequences of the selected vaccine candidate peptides were loaded in one-letter notation in FASTA format. The program predicts whether a peptide is a probable allergen by means of the Tanimoto coefficient, which is used to determine the similarity between molecules; in this case, the physicochemical properties of the amino acids, such as hydrophobicity, size, relative abundance, and probability of forming α-helices or β-folds, were analyzed. The lower this coefficient, the more similar the molecules are.

### Toxicity evaluation of vaccine candidate peptides

To evaluate the toxicity of the vaccine candidate peptides, the ToxinPred server was used [36]; this server compares the sequence of the peptides with those in its database to predict the toxicity of peptides. For this, methods based on SVM (Swiss-Prot) and SVM (TrEMBL) were used, with a cutoff value of 0.0, which is recommended by the server. The peptides that did not show toxicity, as determined by both methods, were selected.

### Search for vaccine candidate peptides in the mature EBOV GP sequence

Vaccine candidate peptides were analyzed to ensure that they are present in mature GP. The sequence used to identify peptides was for immature GP (immature); in the maturation process, certain regions are lost to form the mature and functional GP. Sequence 5JQ3, derived from crystallized mature GP, was downloaded from the PDB, and the peptide sequences were aligned with the mature sequence to ensure that the peptides were present in mature GP.

### Solvent exposure analysis of vaccine candidate peptides within EBOV GP

The ACCPro v5.2 server was used for this analysis [38]. Using a prediction of 1D-RNNs, adopting as an input the multiple alignment of homologs generated by PSI–BLAST, ACCPro correctly classifies up to 90% of residues if there is a homolog in the PDB, for example, the corresponding 5JQ3 protein to the crystallized structure of EBOV [39]. Using the PyMol program (PyMOL Molecular Graphics System, v2.4.0 Schrödinger, LLC), the solvent exposure calculation was performed for each of the candidate peptides within the trimeric GP, for which the PDB structure 5JQ3, a Solvent_radio of 1.4 Å, and a density value of 4 were used to provide the best prediction accuracy. Only candidate peptides present in the mature protein were analyzed using this method.

### Determination of glycosylation sites of GP

To determine the glycosylation sites of GP, the NetNGly v1.0 server was used [40]; this server detects N-glycosylation sites in human proteins using neural networks in the surrounding sequence context to discriminate between acceptor and nonacceptor sequences. Because the Asn-Xaa-Ser/Thr sequence will not always be modified even though it is necessary for glycosylation, the precision of the results generated by the server is 76%. The consensus sequence was analyzed on the server using a cutoff value of 0.5, and the results verified that the candidate peptides were not found in glycosylation sites of GP.

### Antigenicity analysis of vaccine candidate peptides

The vaccine antigenic peptides present in mature GP protein and with solvent exposure greater than 500 Å2 were evaluated using the EMBOSS server. This server uses the method developed by Kolaskar and Tongaonkar to predict antigenic determinants using the physicochemical properties of amino acid residues and their frequencies of appearance in experimentally known epitopes. This algorithm has an accuracy of approximately 75%, and the server identifies regions that have an antigenicity value greater than 1; the higher the value, the greater is the antigenicity [18].

### Docking of vaccine candidate peptides with HLA-A2, A3 and B7

The structures of vaccine candidate peptides 3, 4, 5, 10 and 11 were determined using the PEP-FOLD 3.5 server [41] in which sOPEP energy models were ordered; these models are more effective in identifying peptide-protein complexes. Lower energy sOPEP structures were considered the most stable structures and later used for docking. HLA-A2, HLA-A3 and HLA-B7 were downloaded from the PDB database with the codes 1HHH for A2, 3RL2 for A3 and 5WMN for B7. These structures were coupled to a peptide that was removed by means of the PyMOL program, using AutoDock tools v1.5.7 to obtain only the HLA structures [42]. The HLAs were prepared by adding only polar hydrogens and using a grid box of 40x40x40 points, with the center of the box in the active site and the peptides forming secondary amino acid structures. Finally, using Vina v1.1.2, docking was performed using the structures created with AutoDock tools and the grid box parameters of each peptide structure with each HLA. As controls, 1HHH, 3RL2 and 5WMN were docked with their respective peptides. The lower the Kcal/mol value, the more stable is the bond.

### Refinement of docking

After the first docking, the 9 amino acids of each vaccine candidate peptide that were linked to the active site of HLA were selected; subsequently, 9 amino acid peptides were generated to perform docking under the same conditions. Peptides containing 9 amino acids were selected because this peptide size is common at the time of antigen presentation with HLA.

## Results

### Consensus sequence of the GP of various EBOV species

From the NCBI database, 2,670 amino acid sequences for EBOV GP were downloaded, of which 2,471 remained after applying the exclusion criteria. From these, a consensus sequence was obtained that was used to identify antigenic peptides. The consensus sequence is shown in S1 File.

### Identification of peptides from the EBOV GP consensus sequence

From the consensus sequence, 26 peptides recognized by B lymphocytes were identified using the following programs: BcePred (https://webs.iiitd.edu.in/raghava/bcepred/bcepred_submission.html) (18 peptides); BepiPred (https://services.healthtech.dtu.dk/service.php?BepiPred-2.0) (7 peptides); and Discotopo (https://services.healthtech.dtu.dk/service.php?DiscoTope-2.0) (1 peptide) (Table 1).

**Table 1:** Peptides from the GP consensus sequence of different EBOV species recognized by B lymphocytes, as identified by different servers.

Server	No. of peptides	Sequence
BcePred	18	VTGILQL
		TSFFLWVIILFQR
		FSIPLGVIHN
		TLQVSDVDKLVCRDKLS
		QLRSVGL
		SGVPPKVVNY
		FPRCRYVHKVSG
		FFSSHPL
		VDNLTYVQLE
		FTPQFLLQLN
		MVQVHSQG
		VSHLTTL
		PVYKLDIS
		DGLICGLRQL
		TCHILGPDCCIEPHD
		QIIHDFVD
		IGVTGVII
		LFCICKFVF
BepiPred - 2.0	7	QLPRDRFK
		TLQVSDVDKLVCRDKLSSTNQ
		EGNGVATDVPSVTKRWGFRSGVPPKVVNYEAGEWAENCYNLEIKKPDGSECLPAAPDGIRGFPR
		SGTGPCAGD
		AAGPLKAENTNTSKSADS
		DWTKNITDKIDQI
		VDKTLPDQGDNDNWWTG
Discotopo - 2.0	1	HDWTKNITDKIDQIIHDFV

For T lymphocytes, 203 peptides for the A2, A3, and B7 supertypes were identified using the following servers: Epijen (http://www.ddg-pharmfac.net/epijen/EpiJen/EpiJen.htm) (102 peptides); NetCTLPan (http://www.cbs.dtu.dk/services/NetCTLpan/) (9 peptides); ANNPRED (http://www.imtech.res.in/raghava/nhlapred/neural.html) (11 peptides); MHCPred (http://www.ddg-pharmfac.net/mhcpred/MHCPred/) (15 peptides); NetMHC (https://services.healthtech.dtu.dk/service.php?NetMHC-4.0) (8 peptides); NetCTL (https://services.healthtech.dtu.dk/service.php?NetCTL-1.2) (39 peptides); and nHLAPred (http://www.imtech.res.in/raghava/nhlapred/) (19 peptides) (Table 2).

**Table 2:** Peptides from the GP consensus sequence of different EBOV species recognized by T lymphocytes, as identified by different servers.

		Sequence		
Server	No. peptides	A2	A3	B7
Epijenv1.0	102	FLLQLNETI	VIALFCICK	GVIIAVIAL
		FTPQFLLQL	QIIHDFVDK	FQRTFSIPL
		LIWKVNPEI	GIYTEGLMH	AVSHLTTLA
		ILFQRTFSI	CIEPHDWTK	LQVSDVDKL
		GVATDVPSV	ETKKNLTRK	HPLREPVNA
		RTFSIPLGV	RTFSILNRK	LGVIHNSTL
		STASDTPPA	GIRGFPRCR	SKSADSLDL
		ALFCICKFV	FTAVSNGPK	TVIYRGTTF
		VIYRGTTFA	CAGDFAFHK	TTQALQLFL
		GLICGLRQL	ATTAAGPLK	MASENSSAM
		LITNTIAGV	GILQLPRDR	RDRFKRTSF
		FQRTFSIPL	ATDVPSVTK	AVSNGPKNI
		QLANETTQA	NTTGKLIWK	TTNEDHKIM
		GVTGVIIAV	STHNTPVYK	RWGGTCHIL
		NTIAGVAGL	FLILPQAKK	LNRKAIDFL
		IMASENSSA	TLQVSDVDK	LANETTQAL
		VIHNSTLQV	NISGQSPAR	EGVVAFLIL
		GLMHNQDGL	AIGLAWIPY	AAVSHLTTL
		QLRSVGLNL	QLPRDRFKR	SPQNYSETA
		FLYDRLAST	VTGILQLPR	VVNYEAGEW
		NITDKIDQI	GFRSGVPPK	LFLRATTEL
		KLGLITNTI	KAENTNTSK	TELRTFSIL
		VTGVIIAVI	RLASTVIYR	GVTGVIIAV
		GLNLEGNGV	VIYRGTTFA	DKLSSTNQL
		VAFLILPQA	TSPQPPTTK	EGIYTEGLM
		MGVTGILQL	VQVHSQGRK	SFFLWVIIL
		NQDGLICGL	EVIVNAQPK	THNTPVYKL
		LLQRWGGTC	LITGGRRTR	KIRSEELSF
		TTGKLIWKV	AFLILPQAK	FAFHKEGAF
		VIIAVIALF	KLSSTNQLR	AVIALFCIC
		CLPAAPDGI	ITGGRRTRR	GVATDVPSV
		WIPYFGPAA	ETIYASGKR	AENCYNLEI
		VIVNAQPKC	EATQVGQHH	QVGQHHRRA
		RGTTFAEGV	KAIDFLLQR	EGAAIGLAW
NetCTLPan	9	ILFQRTFSI	RTFSILNRK	MASENSSAM
		FLLQLNETI	RLASTVIYR	
		ALFCICKFV	STHNTPVYK	
		FLYDRLAST		
		GLICGLRQL		
ANNPRED	11	MGVTGILQL	ILFQRTFSI	ILFQRTFSI
		GVTGILQLP	VIHNSTLQV	VIHNSTLQV
		VTGILQLPR	GVATDVPSV	GVATDVPSV
			FLYDRLAST	FLYDRLAST
MHCPred	15	FLLQLNETI	GIYTEGLMH	NGVATDVPS
		LIWKVNPEI	RTFSILNRK	FRSGVPPKV
		GVATDVPSV	FTAVSNGPK	PRDRFKRTS
		VAFLILPQA	TTAAGPLK	RFKRTSFFL
		ILFQRTFSI	RLASVIYR	PSVTKRWGF
NetMHC	8	FLYDRLAST	RTFSILNRK	
		ILFQRTFSI	RLASTVIYR	
		FLLQLNETI	STHNTPVYK	
		YLFEVDNLT		
		ALFCICKFV		
NetCTL	39	ATEDPSSGY	RTFSILNRK	MASENSSAM
		ATTTSPQNY	RLASTVIYR	LANETTQAL
		NSTHNTPVY	FLILPQAKK	RDRFKRTSF
		TEDPSSGYY	STHNTPVYK	AAVSHLTT
		SSDPETNTT	KLSSTNQLR	SPQPPTTKT
			ATTAAGPLK	LPRDRFKRT
			FTAVSNGPK	LPQAKKDFF
			SVTKRWGFR	GPCAGDFAF
			VIALFCICK	IPYFGPAAE
			NTTGKLIWK	SPQNYSETA
			AIGLAWIPY	TVIYRGTTF
			QIIHDFVDK	SASSGKLGL
			ITGGRRTRR	KIRSEELSF
			ATDVPSVTK	
			GYYSTTIRY	
			TSPQPPTTK	
			VQVHSQGRK	
			KAENTNTSK	
			GIYTEGLMH	
			FLRATTELR	
			TQALQLFLR	
nHLAPnet	19	GIRGFPRCR	STHNTPVYK	LPRDRFKRTSF
		LYDRLASTV	EVIVNAQPK	FQRTFSIPL
		GLICGLRQL		GPCAGDFAF
		HILGPDCCI		TVIYRGTTF
		ALFCICKFV		LPQAKKDFF
				DPSSGYYST
				TIRYQATGF
				AAVSHLTTL
				SPQPPTTKT
				IGLAWIPYF
				GPAAEGIYT
				GVIIAVIALF

### Determination of vaccine candidate peptides from multiple alignment with the consensus sequence

In the multiple alignment shown in S2 File, performed between the consensus sequences of the GP protein, it was possible to identify a total of 12 candidate vaccine peptides that met the selection criteria. All candidate peptides have regions that are recognized by B lymphocytes, and for peptides 2, 3 and 12, all the HLA supertypes were also evaluated. The rest of the peptides are recognized by B lymphocytes and HLA supertypes (Table 3).

**Table 3:** Vaccine candidate peptides selected from multiple alignment and their ability to interact with B lymphocytes and HLA supertypes.

						Supertype of T lymphocyte		
Peptide ID	Sequence	Start position	Final position	length (aa)	lymphocytes B	A2	A3	B7
1	MGVTGILQLPRDR	1	13	13	P	P	P	NP
2	TSFFLWVIILFQRTFSIP	17	34	18	P	P	P	P
3	FSIPLGVIHNSTLQV	31	45	15	P	P	P	P
4	TLQVSDVDKLVCRDKLSSTNQL	42	63	22	P	NP	P	P
5	FTPQFLLQLNETI	248	260	13	P	P	NP	NP
6	MVQVHSQGRK	350	359	10	P	NP	P	NP
7	RKAAVSHLTTLATISTSPQ	358	376	19	P	NP	NP	P
8	NSTHNTPVYKLDIS	386	399	14	P	NP	P	P
9	AAGPLKAENTNTSKSADS	426	443	18	P	NP	P	NP
10	DGLICGLRQL	552	561	10	P	P	NP	NP
11	EPHDWTKNITDKIDQIIHDF	611	630	20	P	NP	P	NP
12	GIGVTGVIIAVIALFCICK	654	672	19	P	P	P	P

P =  has regions recognized by B lymphocytes or HLA supertypes A2, A3 and B7 of T lymphocytes

NP =  does not have regions recognized by B lymphocytes or HLA supertypes A2, A3 and B7 of T lymphocytes.

### Analysis of toxicity and allergenicity of vaccine candidate peptides

The analysis of the 12 peptides by the ToxinPred server did not reveal toxicity for any peptide using a cutoff value of 0.0. The toxicity values were -1.89 and -2.47 for peptide 1, which was considered the least toxic, and -0.71 and -0.32 for peptide 10, which was considered the most toxic. The AllergenFP server identified peptides 10 and 11 as possible allergens; despite these results, peptides 10 and 11 were retained in the remaining analyses (Table 4).

**Table 4:** Degree of toxicity and prediction of allergenicity of the candidate peptides.

	ToxinPred					
	SVM (Swiss-Prot)		SVM (TrEMBL)		AllergenFP	
Peptide ID	Prediction	Toxicity	Prediction	Toxicity	Prediction	Tanimoto index
1	Non-Toxin	-1.89	Non-Toxin	-2.47	NON-ALLERGEN	0.58
2	Non-Toxin	-0.97	Non-Toxin	-1.27	NON-ALLERGEN	0.68
3	Non-Toxin	-1.17	Non-Toxin	-1.58	NON-ALLERGEN	0.63
4	Non-Toxin	-0.74	Non-Toxin	-1.04	NON-ALLERGEN	0.71
5	Non-Toxin	-1.13	Non-Toxin	-1.01	NON-ALLERGEN	0.65
6	Non-Toxin	-0.96	Non-Toxin	-1.8	NON-ALLERGEN	0.7
7	Non-Toxin	-1.37	Non-Toxin	-1.52	NON-ALLERGEN	0.66
8	Non-Toxin	-1.09	Non-Toxin	-1.16	NON-ALLERGEN	0.68
9	Non-Toxin	-1.14	Non-Toxin	-0.97	NON-ALLERGEN	0.63
10	Non-Toxin	-0.71	Non-Toxin	-0.32	PROBABLE ALLERGEN	0.67
11	Non-Toxin	-1.54	Non-Toxin	-1.58	PROBABLE ALLERGEN	0.68
12	Non-Toxin	-0.7	Non-Toxin	-0.6	NON-ALLERGEN	0.61

### Exposure to the solvent for the vaccine candidate peptides within the GP of EBOV, as determined by ACCPro

The peptides with the highest exposure to the solvent, as determined by ACCPro, were 1, 4, 6, 7, 8, 9, 11 and 12; peptides 2, 3, 5 and 10 had regions that were not exposed. The peptides that are present in mature GP were 3, 4, 5, 10 and 11, of which 2 presented greater exposure to the solvent, as determined using the PyMol program: peptides 11 (1,559.126 Å^2^) and 4 (1,293.63 Å^2^). The peptide with the lowest exposure to the solvent was peptide 10 (186 Å^2^) (Table 5).

**Table 5:** Analysis of solvent exposure of the vaccine candidate peptides (ACCPro server).

Peptide ID	Sequence	Solvent exposure (ACCPro)	Presence in the mature GP	Solvent exposure PyMol (Å2)
1	MGVTGILQLPRDR	eeee-e-e-eeee	No	–
2	TSFFLWVIILFQRTFSIP	------------ee-e--	No	–
3	FSIPLGVIHNSTLQV	-e------eeee-e-	Yes	626.658
4	TLQVSDVDKLVCRDKLSSTNQL	e-e-ee-ee-eee-e-eeee--	Yes	1,293.63
5	FTPQFLLQLNETI	-e-e--ee-eee-	Yes	510.16
6	MVQVHSQGRK	--e-eeeeee	No	–
7	RKAAVSHLTTLATISTSPQ	eeee-ee-eee-e-eeeee	No	–
8	NSTHNTPVYKLDIS	eeeeee-e-e-e-e	No	–
9	AAGPLKAENTNTSKSADS	eee-eeeeeeeeeeeeee	No	–
10	DGLICGLRQL	ee--------	Yes	186.735
11	EPHDWTKNITDKIDQIIHDF	e-eeeeeeeeeeeeeeeeee	Yes	1,559.126
12	GIGVTGVIIAVIALFCICK	ee-ee-eeeeeeeeeeeee	No	–

“e” =  amino acid exposed to the solvent

“-” =  amino acid without solvent exposure

### Determination of glycosylation sites in the sequences of the vaccine candidate peptides within EBOV GP

The results obtained using the NetNGly server are shown in Table 6. Peptides 3, 5, 8 and 9 had glycosylation sites with a potential greater than 0.5, which is the cutoff value that was used. Peptide 11 did not have a glycosylation site because the potential was less than the cutoff value; the other peptides did not have glycosylation sequences.

**Table 6:** Glycosylation sites present in the vaccine candidate peptides.

Peptide ID	Sequence	Start position	Final position	Glycosylation	Potential
1	MGVTGILQLPRDR	1	13	No	–
2	TSFFLWVIILFQRTFSIP	17	34	No	–
3	FSIPLGVIHNSTLQV	31	45	Yes	0.75
4	TLQVSDVDKLVCRDKLSSTNQL	42	63	No	–
5	FTPQFLLQLNETI	248	260	Yes	0.721
6	MVQVHSQGRK	350	359	No	–
7	RKAAVSHLTTLATISTSPQ	358	376	No	–
8	NSTHNTPVYKLDIS	386	399	Yes	0.6738
9	AAGPLKAENTNTSKSADS	426	443	Yes	0.5853
10	DGLICGLRQL	552	561	No	–
11	EPHDWTKNITDKIDQIIHDF	611	630	No	0.4541
12	GIGVTGVIIAVIALFCICK	654	672	No	–

### Predicted antigenicity of vaccine candidate peptides

The most antigenic vaccine candidate peptide was 4 (1.157), followed by 3, 5 and 11, with the latter having the lowest antigenicity (1.053) (Table 7).

**Table 7:** Antigenicity values for the vaccine candidate peptides.

Peptide ID	Sequence	Antigenic region	Antigenicity
3	FSIPLGVIHNSTLQV	PLGVIHNST	1.14
4	TLQVSDVDKLVCRDKLSSTNQL	VSDVDKLVCRDK	1.157
5	FTPQFLLQLNETI	QFLLQLN	1.134
11	EPHDWTKNITDKIDQIIHDF	KIDQII	1.053

### HLA docking with vaccine candidate peptides, as determined by PEP-FOLD

The PEP-FOLD 3 server predicted 3 structures for peptides 3, 4 and 10, 4 structures for peptide 11 and 5 structures for peptide 5. The interaction energies for peptide-HLA binding (kcal/mol) are shown in Table 8; the most stable binding was between peptide 11 and HLA-A3, with a value of -4.3 kcal/mol, followed by peptides 5 and 10 with HLA-B7, with a value of -4.2 kcal/mol.

**Table 8:** Binding energies between structures of the antigenic candidate peptides and HLAs, as determined by docking.

	HLA		
	A2 (kcal/mol)	A3 (kcal/mol)	B7 (kcal/mol)
PEP3	-4.1	-3.9	-4.1
PEP4	-4.0	-3.8	-3.9
PEP5	-3.8	-3.8	-4.2
PEP10	-4.0	-3.8	-4.2
PEP11	-3.9	-4.3	-3.8
PDB ID Controls	1HHH -8.0	3RL2 -7.2	5WMN -7.7

After refinement of the docking, the most stable binding was between PEP10 and PEP3 with HLA-A3, with binding energies of -7.7 kcal/mol and -7.6 kcal/mol, followed by PEP3 and PEP5 with HLA-B7, with a binding energy of -7.2 kcal/mol; the least stable binding was between PEP11 and HLA-B7, with a binding energy of -5.8 kcal/mol, and between PEP5 and HLA-A3, with a binding energy of -6.3 kcal/mol (Table 9).

**Table 9:** Binding values determined by docking between the peptides (9 amino acids) of vaccine candidate peptides and HLAs.

	HLA		
	A2 (kcal/mol)	A3 (kcal/mol)	B7 (kcal/mol)
PEP3	-6.4	-7.6	-7.2
PEP4	-6.5	-6.7	-6.5
PEP5	-6.8	-6.3	-7.2
PEP10	-6.6	-7.7	-6.6
PEP11	-6.4	-6.5	-5.8
PDB ID Controls	1HHH -8.0	3RL2 -7.2	5WMN -7.7

**Fig. 2:**
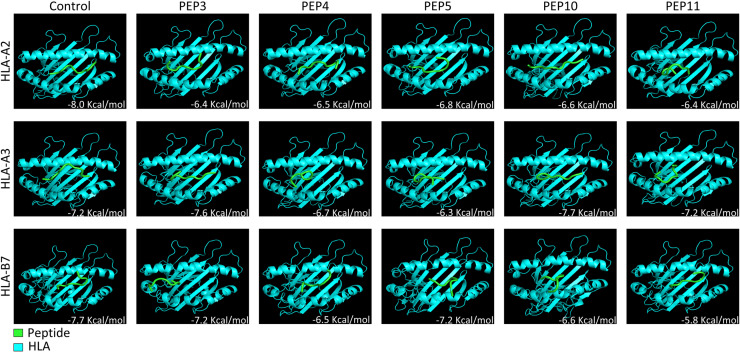
Images of the docking between HLA-A2, A3 and B7 (cyan) with the vaccine candidate peptides (green) indicate the best binding between PEP10 and PEP3 with HLA-A3. The image show the peptides in the HLA groove (red arrows). The peptides with the worst binding were PEP11 with HLA-B7 and PEP5 with HLA-A3; only a part of the peptides is within the HLA groove (purple arrow).

Fig. 2 shows images of the docking that corroborate the aforementioned results. PEP10 and PEP5 are embedded in the groove (active site) of HLA-A3, and therefore, their binding is much more stable; in contrast, only a small region of PEP5 is found within the HLA-B7 groove, with the same occurring in the interaction between PEP5 and HLA-A3.

### Conservation analysis between species and time of the GP regions from which the candidate peptides were determined

**Fig. 3:**
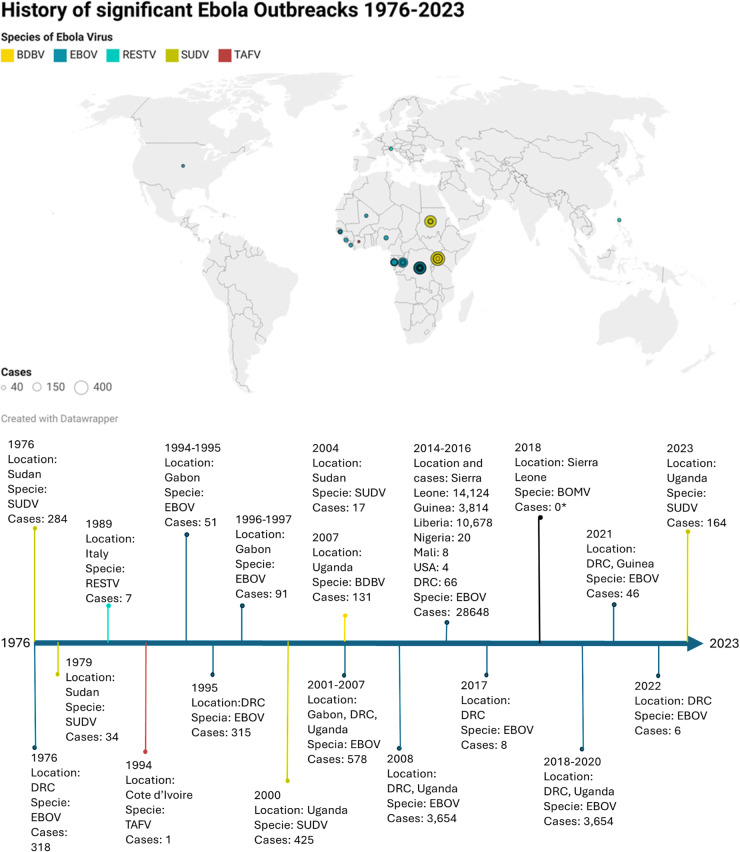
Ebola epidemics from 1973 to 2023. A map shows the number of Ebola cases in different countries around the world. In the cases of Europe and the United States, these are imported cases of people who went to attend an epidemic on the African continent and returned to their countries of origin where they began to show symptoms of the disease. The EBOV species causing the outbreaks are represented in different colors. At the bottom is a timeline with the different outbreaks and epidemics of the disease.

In order to determine the impact of the protection of the determined vaccine candidate peptides, an analysis was carried out taking into account the sequences used to obtain the consensus sequence. In Fig 3 we can observe the EBOV outbreaks from 1976 to 2023 where the sequences used in this work come from, being mainly in the African continent, mainly the Dominican Republic of the Congo where most of the cases come from, added to this the largest outbreak of the disease in 2014-2016 by the Ebola Zaire species affecting Sierra Leone and Nigeria. When comparing the regions where the candidate peptides come from, it was determined that they are stable in terms of time and virus species.

## Discussion

EBOV infection continues to be a major health problem [43]. The highest number of cases occurred between 2014 and 2016 on the African continent, with approximately 28,000 cases and 11,000 deaths. A latency phase is also being studied in this disease; if confirmed, people who have recovered from the disease have the ability to start outbreaks in places with a high population density. Therefore, the best way to control future outbreaks and even EBOV pandemics is through the use of vaccines [44].

Peptide-based vaccines have advantages over other platforms; for example, they can be used in immunocompromised people, unlike live vaccines, where there is a risk of reversing and producing the disease [45]. Another advantage of using only a fragment of GP for EBOV in particular is that the risk of the immunomodulatory effect of complete GP is eliminated, as are the adverse effects [46], in addition to reduced production costs and problems meeting demand [47]. Bioinformatics tools have reduced the costs and time for the development of vaccines; for example, the use of these tools facilitated the rapid control of SARS-CoV-2 [48–50].

Lately, the use of bioinformatics tools represents an advantage in the design of peptide-based vaccines [51], these are: the vaccines have proven to be effective, the development cost decreases, they are precise, robust, safe and harmless to humans [52,53], even some candidate vaccines have entered clinical studies immediately, allowing rapid development and that they can be used in response to emerging diseases [54] as in the case of SARS-CoV-2 [55] and mammarenavirus [56]. Immunoinformatics tools also allow for a robust analysis of etiological agents by being able to analyze the complete genome, the structure and sequence of proteins, something that cannot be done using conventional vaccine development techniques. It has even been possible to develop vaccines against hypervariant agents such as HIV [57,58].

It is important to consider that peptide-based vaccines must meet certain characteristics based on the knowledge of the functioning of the immune system. To do so, it is recommended to follow certain algorithms that were evaluated in this work [59,60].

Using bioinformatic tools, vaccine candidate peptides present in regions that are recognized by B lymphocytes were identified and analyzed. Kurosaki et al. [61] noted that B lymphocytes play an important role in combating viral infections because they are responsible for the production of specific antibodies. Additionally, Kumar et al. [62] noted the important participation of T lymphocytes. In this study, HLA supertypes A2, A3 and B7 were used as they occur in 86% of the world population [27]. Furthermore, Sakabe et al. [63] noted the importance of CTL activation and the important role it play in combating EBOV infections.

**Fig. 4:**
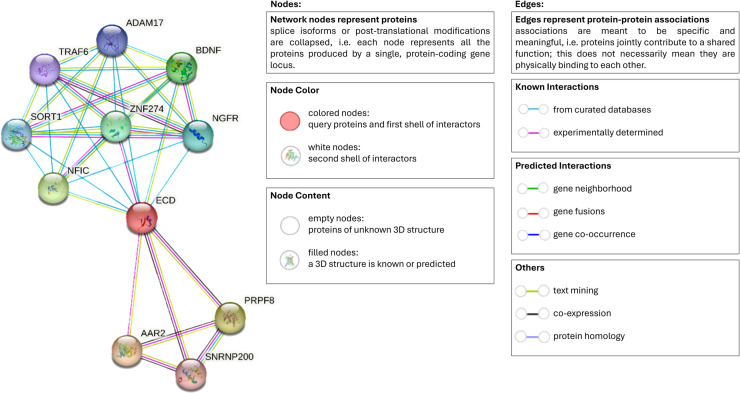
Relationship between AAR2 and ECD (black arrows) determined using the String server ( https://string-db.org/). The relationship between these two proteins was determined experimentally (purple lines).

In this study, 5 vaccine candidate peptides were analyzed. PEP3 (FSIPLGVIHNSTLQV), which is located at positions 31-45 of GP, has a region (FSIPLGVIHN) recognized by B lymphocytes, as determined by the BcePred server *in silico*. In other studies, such as that by Ripoll et al. [64], who analyzed peptide P36 (GVIHNSTLQ), i.e., AA36-44 of GP; this peptide was identified after analyzing which regions of GP are recognized by the antibodies produced by the administration of Ebola Zaire GP to Macaca fascicularis and Mus musculus. Ehrhardt et al. [65] identified a peptide (IPLGVIHNSTLQVSDVD), i.e., AA33-49 of Ebola Zaire GP, that is recognized by antibodies from people who survived Ebola in 1995. This group of antibodies is known as KZ52, and the group of antibodies that recognize various discontinuous epitopes of GP is known as mAb100. In this study, PEP3 shared 13 of the 17 amino acids with the aforementioned peptide, representing 76.5% of its length. Regarding the evaluation of T lymphocytes, PEP3 shared 11 of the 14 amino acids of the peptide EB-GP-Z-10 (LGVIHNSTLQVSDV) identified by Powlson et al. [66] using ELISPOT in human peripheral blood mononuclear cells in which IFNγ elicited these peptides; these cells were obtained from people who received two vaccines, one recombinant and one multivalent (MVA-BN-Filo). Although the regions determined *in silico* to be recognized by B and T lymphocytes are not exactly the same as those seen in other studies, PEP3 could have the ability to stimulate B and T lymphocytes. According to the Immune Epitope Database and Analysis Resource (IEDB), PEP3 shares 5 of the 9 amino acids with a peptide that belongs to the AAR2 protein, which is involved in cancer of the brain (glioblastoma) and ankylosing. This protein is related to the ECD protein, which is a regulator of the stability and function of 53/TP53 and inhibits the degradation of p53/TP53. This relationship was determined experimentally (Fig. 4).

PEP4 (TLQVSDVDKLVCRDKLSSTNQL) is the largest of the vaccine candidate peptides and ranges from AA42-63 of GP. This peptide shares 81.5% homology with the sequence of a peptide identified through an analysis of human antibodies in individuals who showed no symptoms and survived the disease [67]. The peptide ranges from AA41-67 (STLQVSDVDKLVCRDKLSSTNQLRSVG) and was identified with P41 (STLQVSDVDKLVCRDKLSSTNQLRS), i.e., AA41-65 of GP, when analyzing the mAbs c4G7, c2G4, ADI615734 and mAb100. This suggests that this peptide may have the ability to activate B lymphocytes. PEP4 also shares regions with the peptides EB-GP-Z-12 (TLQVSDVDKLVCRDK), EB-GP-Z-13 (SDVDKLVCRDKL) and EB-GP-Z-14 (DVDKLVCRDKLSSTNQL) identified by Powlson et al. [66]. They also have the ability to activate T lymphocytes.

PEP5 (FTPQFLLQLNETI), i.e., AA248-260 of GP, does not share regions with peptides that have the ability to activate certain B lymphocytes in antibodies of people who have recovered from the disease; however, if it shares a region within a peptide identified by Powlson et al. [66]: EB-GP-Z-72 (SRFTPQFLLQLNETI).

PEP3, PEP4 and PEP5 are in the N-terminal region, which has an important function in the process of membrane fusion, as noted by Becquart et al. [67].

PEP10 (DGLICGLRQL), i.e., AA552-561 of GP, is within peptide P544 (TEGLMHNQDGLICGLRQLANETTQALQLFLRATTELRT), i.e., AA544-581 of GP, identified by Ripoll et al. [50] using ELISA and Macaca mulatta sera. It also occurs within a peptide, i.e., AA547-562 (LMHNQDGLICGLRQLA), identified by Sanchez-Lockhart et al. [68] through a western blot of NHP-4672 serum clusters of Macaca fascicularis; only recognized peptides have been reported in antibodies in nonhuman primates, which indicates that PEP10 could have the ability to activate B lymphocytes. Powlson et al. [66] also identified EB-GP-Z-156 (MHNQDGLICGLRQLA) and EB-GP-Z-157 (DGLICGLRQLANETTQA) and suggested that they may have the ability to interact with T lymphocytes, as in the previous cases.

Finally, PEP11 (EPHDWTKNITDKIDQIIHDF), which extends from AA611-630 of GP, shares a sequence with peptides identified by Davis et al. [69] from an analysis of antibodies (ELISA) from 4 donor patients; the peptides ranged from AA611-625 (EPHDWTKNITDKIDQ) and 616-630 (TKNITDKIDQIIHDF), which suggests that PEP11 has the ability to interact with B lymphocytes.

PEP10 and PEP11 occur in a region exclusive to GP and do not share a sequence with sGP, which is an advantage when combating infection because sGP is released by EBOV-infected cells and has a decoy function for EBOV cells through antibodies generated against the N-terminal region of GP, as previously described by Becquart et al. [67]. S1 Table, shows an alignment between the peptides determined in this work and the peptides published in other works mentioned above.

Another important factor in the selection of candidate peptides is safety, for which the toxicity and allergenicity of the candidate peptides are evaluated. For example, scorpions and scorpion venom contain peptides with neurotoxic activity in humans [70]. Regarding allergenicity, it is important to avoid an exacerbated immune response [71], which can translate into adverse effects, as seen with other vaccines [46]. Other studies have employed the ToxinPred server; for example, it was used to evaluate the hemolytic activity of peptides in the study by Robles-Loaiza et al. [72] and was also used to search for peptides in SARS-CoV-2 in the study by Cihan and Ozger [73], in which they evaluated the toxicity of the 62 peptides that they identified, which were all nontoxic, with the highest value of -0.15. In our study, the highest value was -0.32, a value further from the cutoff value, which was 0.0 Thus, the probability that the peptides are toxic is low. Despite the *in silico* prediction of toxicity, it is necessary to validate these results in vitro to avoid unwanted adverse effects of vaccines at the time of use [74].

In this study, exposure of the candidate peptides to the solvent was analyzed, with the goal of ensuring that the antibodies generated from these peptides do not have steric hindrance that keeps them from interacting with the regions of GP from which they come. Additionally, a search for glycosylation sites was conducted, with the aim of ensuring that carbohydrates would not impede recognition sites [75]. The selected peptides were assessed to determine if they occur in mature GP, as GP loses fragments in the maturation process [76]. Many of the peptides found in other studies are not present in mature GP, and thus, the antibodies generated would not recognize GP. Vaccine candidate peptides are present in mature GP, and when compared with the peptides identified from the sera of patients who have recovered from the disease, many of them are exposed on the GP structure.

The binding energies obtained between the HLA and the vaccine candidate peptides ranged from -5.8 to -7.7 kcal/mol, and for the controls, the binding energies ranged from -7.2 to -8.0 kcal/mol. The binding energies of PEP3 with HLA-A3 and B7, PEP5 with HLA-B7 and PEP10 with HLA-A3 were within the range observed for the controls. The values reported by Jain and Baranwal [47] for peptides from EBOV GP and HLA class II molecules ranged from -5.5 to -7.1 kcal/mol and for peptides from EBOV GP and HLA class I molecules ranged from -7.5 to -8.1 kcal/mol. In the study by Montes-Grajales and Olivero-Verbel [77], several peptides from SARS-CoV-2 proteins and several HLAs were evaluated, among which the binding energies between SARS-CoV-2 peptides and HLA-A3 ranged from -5.8 to -8.1 Kcal/mol and those between SARS-CoV-2 peptides and HLA-B7 ranged from -6.7 to -9.4 kcal/mol. Ngo et al. [78] performed ligand‒protein docking and evaluated enzyme inhibitory substances against SARS-CoV-2 using -7 kcal/mol as the cutoff value. These 3 studies used the same program as that used in this study, and most of the binding energies obtained herein were approximately -7 kcal/mol and within the ranges reported in other studies. PEP3, PEP4, PEP5 and PEP10 had values greater than -6.5, and only PEP11 had value below -6.5 against the HLA evaluated here.

PEP4 (TLQVSDVDKLVCRDKLSSTNQL) had favorable results for all the characteristics analyzed in this work: regions recognized by B lymphocytes, regions recognized by the HLA supertypes A3 and B7; no toxicity or allergenicity; present in mature GP; no glycosylation sites; exposed to the solvent; and good binding with the 3 types of HLA.

Although PEP4 has the best characteristics, the other vaccine candidate peptides identified in this study can be used for different platforms, such as the MAP format [79], in nanoparticles [80,81] as VLPs [82,83,84] or to build a polyprotein [85,86]. Various adjuvants that enhance the immune response can also be used, such as XS15 [87]. An advantage of using the vaccine peptide platform is that by using any of the aforementioned strategies, it is possible to rapidly manufacture and distribute the vaccine worldwide in the event of a possible pandemic [88].

In addition to the above, the results obtained from the conservation of the regions from which the candidate peptides come in the sequences that were used to generate the consensus sequence, we can observe that these regions are conserved between the species that have generated outbreaks. In 2014-2016, the largest outbreak in the history of this disease was caused by Ebola Zaire mainly in Sierra Leone, DRC and Nigeria, this led to the authorization of the emergency use of the ERVEBO vaccine, thereby achieving control of the epidemic, however, in 2022-2023 a new outbreak arose due to the SUDV species, seeing the need to generate a vaccine against this other species of the virus. This is where the importance of the candidate peptides determined in this work coming from conserved regions between the species of the virus and over time stands out, where if a similar case were to occur, based on the *in silico* analyses and results, there would be protection against outbreaks produced by the different species and for a longer time.

In this work, an *in silico* study was carried out to find peptides that allow directing the immune response against the complete GP. However, it is important to carry out studies in cell culture to evaluate whether the candidate peptides have the capacity to stimulate antigen-presenting cells and cytotoxicity. Subsequently, it would be important to carry out pre-clinical and clinical tests evaluating the potency of the vaccine, selection of the best adjuvant, route of administration, duration of protection and dose. Finally, a comparison must be made with the reference vaccines approved by health authorities [89–91].

## Conclusions

The vaccine candidate peptides described in this work, especially PEP4, have the ability to interact with B and T lymphocytes, are exposed to the solvent, do not present glycosylation sites, are not toxic or allergenic, are present in mature GP and have good binding with HLA-A2, A3 and B7, as determined from the bioinformatic analyses carried out. These characteristics are supported by other studies that have presented peptides that share sequences. These peptides can be tested in various vaccine platforms for *in vitro* and *in vivo* evaluations of their capacity as vaccine candidates*.*

## Supporting information

S1 File Multiple alignment of consensus sequences.
(TXT)

S2 File Multiple alignment.
(TXT)

S1 Table Alignment of the peptides determined in this work those published in other articles. (DOCX)
