# Supplementary material for: In silico design of Ebola virus Glycoprotein antigenic peptides as vaccine candidates
Source: PLoS One. 2025 Mar 28;20(3):e0319496. doi: 10.1371/journal.pone.0319496 (PMC11952221; doi:10.1371/journal.pone.0319496)
Supplement: S1 Table — (DOCX) [file pone.0319496.s003.docx]

**Table 10. Alignment of the peptides determined in this work with those published in other articles.**

| ID peptide | Sequence | Articles |
| --- | --- | --- |
| PEP3 | FSIPLGVIHNSTLQV---- | This work |
|  | -----GVIHNSTLQ----- | Ripoll et al. [54] |
|  | --IPLGVIHNSTLQVSDVD | Ehrhardt et al. [55] |
|  | ----LGVIHNSTLQVSDV- | Powlson et al. [56] |
|  |  |  |
| PEP4 | -TLQVSDVDKLVCRDKLSSTNQL---- | This work |
|  | STLQVSDVDKLVCRDKLSSTNQLRSVG | Becquart et al. [57] |
|  | STLQVSDVDKLVCRDKLSSTNQLRS-- | Powlson et al. [56] |
|  | -TLQVSDVDKLVCRDK----------- | Powlson et al. [56] |
|  | -----SDVDKLVCRDKL---------- | Powlson et al. [56] |
|  | ------DVDKLVCRDKLSSTNQL---- | Powlson et al. [56] |
|  |  |  |
| PEP5 | --FTPQFLLQLNETI | This work |
|  | SRFTPQFLLQLNETI | Powlson et al. [56] |
|  |  |  |
| PEP10 | --------DGLICGLRQL-------------------- | This work |
|  | TEGLMHNQDGLICGLRQLANETTQALQLFLRATTELRT | Ripoll et al. [50] |
|  | ---LMHNQDGLICGLRQLA------------------- | Sanchez-Lockhart et al. [58] |
|  | ----MHNQDGLICGLRQLA------------------- | Powlson et al. [56] |
|  | --------DGLICGLRQLANETTQA------------- | Powlson et al. [56] |
|  |  |  |
| PEP11 | EPHDWTKNITDKIDQIIHDF | This work |
|  | EPHDWTKNITDKIDQ----- | Davis et al. [59] |
|  | -----TKNITDKIDQIIHDF | Davis et al. [59] |
